# Report of the Prime Minister's Committee

**Published:** 1918-05-18

**Authors:** 


(*To be continued.*)

### THE POSITION OF SCIENCE IN EDUCATION.

### REPORT OF THE PRIME MINISTER'S COMMITTEE.

### II. The Choice of a Profession: English, Natural Science, and Mathematics.

At sixteen, according to the recommendation of the Committee, a boy may leave school, but he need not do so. Many lads, especially at the great public schools, remain until eighteen, and even nineteen; and not until he is eighteen can he register as a medical student. It is therefore advantageous for him, if he looks forward to a medical career, to remain at school till he is eighteen.

The choice of his profession will or should be made on attaining the age of sixteen. By that age his inclinations and aptitudes will usually be sufficiently developed to allow of a definite choice of a profession being made, and from that age his education will no longer be a purely general liberal education adapting him to any pursuit towards which he may incline, but will take on a certain specialisation. He will still study much the same subjects as before, but he will study them in their relations to the profession that he has chosen. In the chemistry course, for instance, all the lads will still study the properties of nitrogen, but the future agriculturist will study it as an ingredient in manures and a necessary condition for the growth of plants, the future soldier will study it as an ingredient in explosives, the future chemist will study its chemical combinations, and the future doctor will regard it as a constituent of protein. There will be no separate classes in chemistry for the different classes of aspirants, but the aspirants to the several professions will be guided along different lines.

It is by no means intended that the two years between sixteen and eighteen should be devoted wholly to natural science, even by those who look forward to a scientific career. Their other studies are to be continued. History, English, some other language, geography, and a choice of other subjects are still to be studied, and at this stage, as at every preceding stage, the study of mathematics is to go hand in hand with the study of natural science; but specialisation of studies is begun. It is begun not only by the direction of attention to those aspects of science that have a direct bearing on subsequent studies, but also by the apportionment of more time to the study of science by those who are to adopt a scientific profession. The proposal is so manifestly sensible, right, and proper that it is sure to evoke the strongest opposition from the worshippers of things as they are, and especially from the public schools.

#### The General Medical Council's Action.

For this change the way is prepared; nay, it is in fact actually effected. A part of the last two years spent at a school where the teaching of natural science is satisfactory is now allowed by the General Medical Council to count as part of the five years that must be spent in medical education before a qualification is obtained. At some schools these preliminary subjects of the medical course are admirably taught. The pupil leaves school with a good knowledge of physics and chemistry, and sometimes even with a groundwork of biology. There is no reason why this modicum of biology should not be increased, time for it being found by dropping out some subject of the earlier school course. The first professional examination usually consists of two groups of subjects, (*a*) physics and chemistry, (*b*) biology, which may be taken separately. It is of manifest advantage to the student, and through him to the profession of medicine, and through that to the country at large, that the student, if he is able to do so, should have passed in at least one of these groups before entering the university or the medical school, and should be able to devote the whole or the greater part of his first year at this institution to his medical studies proper, instead of occupying it, as many now do, in cramming a very superficial and perfunctory knowledge of the preliminary subjects, a knowledge sufficient to enable him to scrape through his examination on the subjects, but utterly insufficient to vivify his medical studies or to prepare his mind for the reception of medical knowledge. Yet at some universities, the student, however satisfactory his previous knowledge of these subjects may be, is compelled to pass the first year of his medical course in going over the ground again, and thus to diminish by one-fifth the time that he might spend, and should spend, in the study of anatomy, physiology, and the more advanced subjects of medicine. Sir Clifford Allbutt,

☛ The previous article appeared on May 11. p. 117.

**POSITION OF SCIENCE IN EDUCATION—(*cont.*).**

the Regius Professor of Physic at Cambridge, where the plan proposed is already in force, and students are allowed to take their first professional examination on entering the university, says there has been a considerable rise of standard since the change was made.

A Ridiculous Provision.

That a knowledge of Greek should be compulsory upon students entering a university is now insisted upon only by country parsons and other fossils who have long been out of touch with education, but these are sufficiently numerous to maintain this ridiculous provision at the old universities. As a rule, after obtaining an entrance scholarship in science at Oxford or Cambridge, a boy begins to cram enough Greek to enable him to pass Responsions or the Previous Examination. The last term at school before entering the university ought to play an important part in a boy's intellectual development, for he then has time to widen his outlook by independent thought and study, and thus correct some of the limitations of his reading which are inevitable in the time during which he is preparing for a scholarship competition. This opportunity is denied to him if he has to take up the superficial study of Greek, revert to work of a lower standard and more elementary grade than any that he has pursued for years, and cram enough Greek to enable him to scrape through the examination. Even greater harm is done to a boy who, though not up to the scholarship standard, is quite capable of making good use of the opportunities for the study of science afforded by the university. "We are keenly alive," says the Report, "to the necessity for developing the literary training of the student of science. This object is certainly not obtained by causing him to learn by heart a translation of a set book and a portion of Greek grammar." It is difficult to suppose that even a pedant can think that it is.

But the Committee goes further than to scout the necessity for compulsory Greek. Its Report deprecates the compulsory imposition of Latin also. The General Medical Council professes not to register a student unless he passes in Latin; but the Council accepts for registration the matriculation examinations of the universities of London, Liverpool, Leeds, Sheffield, and Birmingham, although Latin is not a compulsory subject in any of them. Thus a student may pass on at any of these universities to the highest medical qualifications without showing that he has ever learnt a word of Latin. The Committee therefore considers that, after the door has been opened in this way, it is too late to attempt to retain Latin as an obligatory subject in the examination for entrance into the medical profession. So perishes a hoary superstition, not by any direct assault, but by inanition from neglect.

The First School Certificate, granted after examination at the age of sixteen, should be a sufficient guarantee that the captor of the certificate has received a liberal education fitting him to enter a liberal profession. The Committee proposes that the certificate should be given in several forms, one of which will show merely that the holder has reached a certain standard in the First School Examination, while another shall show in addition that he has attended continuously at a secondary school, and has taken advantage of the opportunities therein afforded, and a third shall show that he has achieved more than a mere pass. It is this third form of certificate that should open the door of the universities and professions to the scholar, and it is proposed by the Committee that this certificate should not be awarded unless the candidate has shown a reasonable amount of attainment in (1) English subjects, (2) languages, (3) mathematics and natural science. A fourth section of subjects is of a miscellaneous character, and includes drawing, music, manual instruction, housecraft, and so forth, but it is not proposed that failure in this section should disqualify a candidate from entrance into a profession or university. While it is proposed that this should be the ordinary portal to the professions, including that of medicine, it is not proposed that it shall be the sole portal. For exceptional cases exceptional means are provided.

Neglect of the English Language.

On certain points the Committee is very insistent, and, in our opinion, very rightly insistent. Throughout the Report stress is laid upon the great importance of a sufficient mastery of the English language. Again and again the Committee recurs to this recommendation, and, in truth, it cannot be too strongly insisted upon, whether from its own intrinsic importance or from the scandalous neglect that it now receives. The English of writers upon science is, as a rule, bad, very bad; but it is a well of English pure and undefiled in comparison with the English of those who have been educated on the Classical Side, and especially of Government officials; and the very worst specimens of English that disgrace their producers are those of the classical schoolmaster. It appears that the use of the English language is not taught in schools, perhaps because there is no schoolmaster competent to teach it; and if, as is to be supposed, the boys model their English on the examples set before them by their masters, it is not to be wondered at that the standard is as disgracefully low as it generally is.

Another matter on which the Committee insists strongly, and in which we are in full agreement with it, is the coupling of natural science with mathematics. The Committee insists that the two subjects are germane to one another, that the chief use of mathematics is in its application to natural science, and that natural science cannot be made exact without the aid of mathematics. Hence it advocates the teaching of both subjects in the early stages by the same teacher, and hence it deprecates the regulation now in force, or proposed by the Board of Education, that a pass may be attained by proficiency in mathematics alone, without any knowledge on the part of the candidate of natural science. *(To be continued.)*

